# Supplementary material for: Iterative improvement in the automatic modular design of robot swarms
Source: PeerJ Comput Sci. 2020 Dec 7;6:e322. doi: 10.7717/peerj-cs.322 (PMC7924708; doi:10.7717/peerj-cs.322)
Supplement: Supplemental Information 3 [file peerj-cs-06-322-s003.zip › argos3/doc/api/standalone/a00534_source.html]

ARGoS: plugins/robots/generic/simulator/positioning\_default\_sensor.cpp Source File


- Main Page
- Related Pages
- Namespaces
- Classes
- Files

- File List
- File Members

# plugins/robots/generic/simulator/positioning\_default\_sensor.cpp

Go to the documentation of this file.

```
00001 
00007 #include <argos3/core/simulator/simulator.h>
00008 #include <argos3/core/simulator/entity/embodied_entity.h>
00009 #include <argos3/core/simulator/entity/composable_entity.h>
00010 
00011 #include "positioning_default_sensor.h"
00012 
00013 namespace argos {
00014 
00015    /****************************************/
00016    /****************************************/
00017 
00018    CPositioningDefaultSensor::CPositioningDefaultSensor() :
00019       m_pcEmbodiedEntity(NULL),
00020       m_pcRNG(NULL),
00021       m_bAddNoise(false) {}
00022 
00023    /****************************************/
00024    /****************************************/
00025 
00026    void CPositioningDefaultSensor::SetRobot(CComposableEntity& c_entity) {
00027       m_pcEmbodiedEntity = &(c_entity.GetComponent<CEmbodiedEntity>("body"));
00028       m_sReading.Position = m_pcEmbodiedEntity->GetOriginAnchor().Position;
00029       m_sReading.Orientation = m_pcEmbodiedEntity->GetOriginAnchor().Orientation;
00030    }
00031 
00032    /****************************************/
00033    /****************************************/
00034 
00035    void CPositioningDefaultSensor::Init(TConfigurationNode& t_tree) {
00036       try {
00037          CCI_PositioningSensor::Init(t_tree);
00038          /* Parse noise range */
00039          GetNodeAttributeOrDefault(t_tree, "pos_noise_range", m_cPosNoiseRange, m_cPosNoiseRange);
00040          GetNodeAttributeOrDefault(t_tree, "angle_noise_range", m_cAngleNoiseRange, m_cAngleNoiseRange);
00041          GetNodeAttributeOrDefault(t_tree, "axis_noise_range", m_cAxisNoiseRange, m_cAxisNoiseRange);
00042          if(m_cPosNoiseRange.GetSpan() != 0 ||
00043             m_cAngleNoiseRange.GetSpan() != CRadians::ZERO ||
00044             m_cAxisNoiseRange.GetSpan() != 0) {
00045             m_bAddNoise = true;
00046             m_pcRNG = CRandom::CreateRNG("argos");
00047          }
00048       }
00049       catch(CARGoSException& ex) {
00050          THROW_ARGOSEXCEPTION_NESTED("Initialization error in default positioning sensor", ex);
00051       }
00052    }
00053 
00054    /****************************************/
00055    /****************************************/
00056    
00057    void CPositioningDefaultSensor::Update() {
00058       m_sReading.Position = m_pcEmbodiedEntity->GetOriginAnchor().Position;
00059       if(m_bAddNoise) {
00060          m_sReading.Position += CVector3(m_pcRNG->Uniform(m_cPosNoiseRange),
00061                                          m_pcRNG->Uniform(m_cPosNoiseRange),
00062                                          m_pcRNG->Uniform(m_cPosNoiseRange));
00063          m_pcEmbodiedEntity->GetOriginAnchor().Orientation.ToAngleAxis(m_cAngle, m_cAxis);
00064          m_cAngle += CRadians(m_pcRNG->Uniform(m_cAngleNoiseRange));
00065          m_cAxis += CVector3(m_pcRNG->Uniform(m_cAxisNoiseRange),
00066                              m_pcRNG->Uniform(m_cAxisNoiseRange),
00067                              m_pcRNG->Uniform(m_cAxisNoiseRange));
00068          m_sReading.Orientation.FromAngleAxis(m_cAngle, m_cAxis);
00069       }
00070       else {
00071          m_sReading.Orientation = m_pcEmbodiedEntity->GetOriginAnchor().Orientation;
00072       }
00073    }
00074 
00075    /****************************************/
00076    /****************************************/
00077 
00078    void CPositioningDefaultSensor::Reset() {
00079       m_sReading.Position = m_pcEmbodiedEntity->GetOriginAnchor().Position;
00080       m_sReading.Orientation = m_pcEmbodiedEntity->GetOriginAnchor().Orientation;
00081    }
00082 
00083    /****************************************/
00084    /****************************************/
00085 
00086    REGISTER_SENSOR(CPositioningDefaultSensor,
00087                    "positioning", "default",
00088                    "Carlo Pinciroli [ilpincy@gmail.com]",
00089                    "1.0",
00090                    "A generic positioning sensor.",
00091                    "This sensor returns the current position and orientation of a robot. This sensor\n"
00092                    "can be used with any robot, since it accesses only the body component. In\n"
00093                    "controllers, you must include the ci_positioning_sensor.h header.\n\n"
00094                    "REQUIRED XML CONFIGURATION\n\n"
00095                    "  <controllers>\n"
00096                    "    ...\n"
00097                    "    <my_controller ...>\n"
00098                    "      ...\n"
00099                    "      <sensors>\n"
00100                    "        ...\n"
00101                    "        <positioning implementation=\"default\" />\n"
00102                    "        ...\n"
00103                    "      </sensors>\n"
00104                    "      ...\n"
00105                    "    </my_controller>\n"
00106                    "    ...\n"
00107                    "  </controllers>\n\n"
00108                    "OPTIONAL XML CONFIGURATION\n\n"
00109                    "It is possible to add uniform noise to the sensor, thus matching the\n"
00110                    "characteristics of a real robot better. You can add noise through the\n"
00111                    "attributes 'pos_noise_range', 'angle_noise_range', and 'axis_noise_range'.\n"
00112                    "Attribute 'pos_noise_range' regulates the noise range on the position returned\n"
00113                    "by the sensor. Attribute 'angle_noise_range' sets the noise range on the angle\n"
00114                    "(values expressed in degrees). Attribute 'axis_noise_range' sets the noise for\n"
00115                    "the rotation axis. Angle and axis are used to calculate a quaternion, which is\n"
00116                    "the actual returned value for rotation.\n\n"
00117                    "  <controllers>\n"
00118                    "    ...\n"
00119                    "    <my_controller ...>\n"
00120                    "      ...\n"
00121                    "      <sensors>\n"
00122                    "        ...\n"
00123                    "        <positioning implementation=\"default\"\n"
00124                    "                     pos_noise_range=\"-0.1:0.2\"\n"
00125                    "                     angle_noise_range=\"-10.5:13.7\"\n"
00126                    "                     axis_noise_range=\"-0.3:0.4\" />\n"
00127                    "        ...\n"
00128                    "      </sensors>\n"
00129                    "      ...\n"
00130                    "    </my_controller>\n"
00131                    "    ...\n"
00132                    "  </controllers>\n\n"
00133                    "OPTIONAL XML CONFIGURATION\n\n"
00134                    "None.\n",
00135                    "Usable"
00136                   );
00137 
00138 }
```

---

Generated on 10 Jul 2018 for ARGoS by 
 1.6.1
